# Crystalline sirolimus-coated balloon (cSCB) angioplasty in an all-comers, patient population with stable and unstable coronary artery disease including chronic total occlusions: rationale, methodology and design of the SCORE trial

**DOI:** 10.1186/s12872-023-03187-x

**Published:** 2023-03-31

**Authors:** Sylvia Otto, Victor Alfonso Jiménez Díaz, Daniel Weilenmann, Florim Cuculi, Amin Ariff Nuruddin, Gregor Leibundgut, Fernando Alfonso, Wan Azman Wan Ahmad, Stylianos Pyxaras, Harald Rittger, Philip Steen, Luise Gaede, Christian Schulze, Jochen Wöhrle, Mark Rosenberg, Matthias W. Waliszewski

**Affiliations:** 1 Department of Internal Medicine I, Division of Cardiology, Pneumology, Angiology and Intensive Medical Care, University Hospital Jena, Friedrich-Schiller-University Jena, Jena, Germany; 2 Hospital Álvaro Cunqueiro, Vígo, Spain; 3 Kantonsspital St Gallen, St Gallen, Switzerland; 4 Luzerner Kantonsspital, Luzern, Switzerland; 5 The National Heart Institute of Malaysia, Kuala Lumpur, Malaysia; 6 Universitätsspital Basel, Basel, Switzerland; 7 Hospital La Princesa, Madrid, Spain; 8 University Malaya Medical Centre, Kuala Lumpur, Malaysia; 9 Klinikum Fürth, Fürth, Germany; 10 Department of Medical Scientific Affairs, Vascular Systems, Aesculap, B. Braun Melsungen AG, Berlin, Germany; 11 Universitätsklinikum Erlangen, Erlangen, Germany; 12 Klinik für Kardiologie, Angiologie, Pneumologie und internistische Intensivmedizin, Klinikum Friedrichshafen GmbH, Friedrichshafen, Germany; 13 Medizinischen Klinik I, Klinikum Aschaffenburg, Aschaffenburg, Germany; 14 Internal Medicine and Cardiology, Charité Universitätsmedizin, Campus Virchow, Berlin, Germany

**Keywords:** Drug-coated balloon, Sirolimus, De novo lesions, In-stent stenosis, Re-stenosis, Percutaneous transluminal coronary angioplasty

## Abstract

**Background:**

A decade ago, the iopromide-paclitaxel coated balloon (iPCB) was added to the cardiologist‘s toolbox to initially treat in-stent restenosis followed by the treatment of de novo coronary lesions. In the meantime, DES technologies have been substantially improved to address in-stent restenosis and thrombosis, and shortened anti-platelet therapy. Recently, sirolimus-coated balloon catheters (SCB) have emerged to provide an alternative drug to combat restenosis.

**Methods:**

The objective of this study is to determine the safety and efficacy of a novel crystalline sirolimus-coated balloon (cSCB) technology in an unselective, international, large-scale patient population. Percutaneous coronary interventions of native stenosis, in-stent stenosis, and chronic total occlusions with the SCB in patients with stable coronary artery disease or acute coronary syndrome were included. The primary outcome variable is the target lesion failure (TLF) rate at 12 months, defined as the composite rate of target vessel myocardial infarction (TV-MI), cardiac death or ischemia-driven target lesion revascularization (TLR). The secondary outcome variables include TLF at 24 months, ischemia driven TLR at 12 and 24 months and all-cause death, cardiac death at 12 and 24 months.

**Discussion:**

Since there is a wealth of patient-based all-comers data for iPCB available for this study, a propensity-score matched analysis is planned to compare cSCB and iPCB for the treatment of de novo and different types of ISR. In addition, pre-specified analyses in challenging lesion subsets such as chronic total occlusions will provide evidence whether the two balloon coating technologies differ in their clinical benefit for the patient.

**Trial registration number:**

ClinicalTrials.gov Identifier: NCT04470934.

## Background

In the early days of interventional cardiology, plain-old balloon angioplasty (POBA) was the only available tool to treat coronary lesions. However, POBA had its limitations such as high restenosis rates in the range of 30–50% and the occurrence of acute and subacute vessel closure [1]. A second milestone was reached when bare metal stents (BMS) were introduced to address the aforementioned POBA limitations. However, in-stent restenosis (ISR) which is basically a foreign-body reaction to a permanent implant, which manifests itself in neointimal hyperplasia. As a consequence, drug-eluting stents (DES) found their way into the interventional ‘toolbox’ to effectively suppress neointimal proliferation and to reduce restenosis rates in the 5–15% range [2]. Despite continuous enhancements of DES technologies, the Achilles heel of DES is still rooted in local hypersensitivity, neointimal hyperplasia, persistent inflammation, neoatherosclerosis and stent thrombosis (ST) provoked by several vascular mechanisms in response to a permanent coronary implant [3, 4]. Even the latest DES improvements do entail that the lesion is ‘encaged’ within a metal implant without the capacity of positive remodeling, i.e. a lumen enlargement, or vasomotion. Vasomotion, vessel pulsatility and physiological vessel angulation are of growing interest since they seem to play a role for future adverse cardiac events [5]. Moreover, for modern DES, very-late stent related events occur following the first year after PCI at a rate of 2% per year and lead to a target-lesion failure rate of approximately 14% after 5 years [6–8].

The appealing concept of a PCI without permanent implants leaves only two options, absorbable stents or drug-coated balloon angioplasty. The attractiveness of absorbable stents was lost with the onset of reports of unacceptably high stent thrombosis rates [9] which enables DCB angioplasty to move closer to the center stage of clinical research.

Nevertheless, pivotal studies for DCB angioplasty were primarily conducted to treat ISR [10]. Numerous studies provided sufficient evidence for a renewed class I recommendation by the European Society of Cardiology for in-stent restenosis with an A evidence level [11]. While initially safety and efficacy studies focused on ISR, de novo lesions were studied with paclitaxel-eluting stents as comparators. The BELLO study [12] randomized DCB and DES with paclitaxel coating to treat small vessel de novo lesions. They found that late lumen loss was lower in the DCB group as compared to DES while the binary restenosis and revascularization rates were similar in both groups. Moreover, the clinical endpoint powered BASKET SMALL II trial in small vessel de novo lesions [13] revealed comparable outcomes for DCB angioplasty and newer generation DES. This growing clinical evidence supports the intuitive attractiveness of DCB angioplasty with its obvious benefit of avoiding coronary foreign body implants [14]. The latter may also help to rationalize a shortened dual antiplatelet therapy (DAPT), in particular for elective patients with de novo lesions.

The drug-coated balloon catheter, used in this study, carries an alternative drug, i.e. sirolimus, as compared to the predecessor devices with an iopromide-paclitaxel coating. The iopromide-paclitaxel coated DCB was extensively studied in several studies, e.g., the SeQuent Please Worldwide 2000 Registry [14], the DCB-only All-comers Registry [15], BASKET SMALL 2 [16] as well as the PASSWORD Registry [17].

There is a certain analogy of searching for the most promising drug to inhibit intimal proliferation following stenting and ballooning. In the early stages of drug-eluting stents (DES) there were two drugs, i.e., paclitaxel and sirolimus which were used for a polymer-matrix based drug release from stent struts. Limus-eluting stents replaced paclitaxel eluting stents over time due to significantly lower rates of target-lesion failure [18–20]. Also, paclitaxel has less favorable, cytotoxic vessel effects by inducing media necrosis and focal wall hemorrhage compared to the cytostatic (siro-) limus drugs [21]. A meta-analysis of paclitaxel-coated devices used for endovascular treatment of peripheral artery disease showed a signal toward higher mortality during long-term follow-up [22]. This finding was not confirmed by several later studies, but remains still a concern and controversy, and led to a reluctance towards paclitaxel-coated balloons with great geographical differences [23, 24]. Despite cardinal differences between stent-mediated and balloon-mediated drug release, it seems the next logic step to study the safety and efficacy in sirolimus-coated balloons in an unselected patient population.

Currently, several technologies are being investigated [25–28] and few publications are highlighting differences of DCB-technologies as well as its clinical options [29, 30].

In this context, Ali et al. [28] compared crystalline sirolimus-coated balloons (cSCB) to iopromide-paclitaxel-coated balloons in patients with in-stent restenosis. The design of the angiographic endpoint trial was such that a small group of patients was necessary to show non-inferiority and underscore the device’s safety. However, as compared to uncoated balloon angioplasty, the anti-proliferative result of sirolimus on the otherwise identical catheter was remarkable, showing no differences in terms of TLR and MACE at 12-month follow-up [31]. Brigouri et al. [32] investigated SCB and PCB angioplasty in in-stent restenosis and concluded that the target lesion failure rates were similar at one year. Additional clinical data comparing paclitaxel and sirolimus coated balloons in particular for de novo lesions have been recently published [33–35].

Bleeding risk under DAPT remains an important clinical issue, especially considering growing PCI volumes in elderly patients. The DCB-only strategy in de novo vessel offers an advantage over stent implantation since the recommended duration of DAPT is only 4 weeks based on the results of clinical trials and expert opinion [25]. Unfortunately, the latest guidelines do not differentiate between DES implantation or drug-coated balloon-only PCI and recommend a default strategy of 6 months DAPT for all stable CAD patients with the option of shortening to 3 months and up to 4 weeks [26].

In summary, the concept of an “implant-free” PCI remains an attractive, feasible and modern treatment option for patients with coronary artery disease and indication for PCI. Sirolimus, as a cytostatic “limus” drug might offer favorable vessel effects compared to the cytotoxic drug paclitaxel, and reveals a much greater therapeutic window. Based on DES data, (siro)limus drugs have a higher anti-restenotic efficacy, also including anti-inflammatory properties. It remains unclear, if and when these differences in drug formulations translate into clinically relevant vessel healing reactions after an “implant-free” PCI.

Also, treatment of CTO lesions with a drug-coated balloon might be beneficial for a subsect of patients in this high-risk cohort. The use of drug-coated balloons, if necessary in combination with stent implantation of a proximal or distal segment of the target vessel can prevent “full-metal-jacket” situations, and thus provide favorable long-term outcomes. Since, evidence of DCB interventions in CTO lesions is scarce, we specifically included these lesions in a predefined subgroup at the operators’ discretion.

Therefore, the rationale of this observational, post-market, single-armed study is to confirm the safety and efficacy of crystalline sirolimus-coated balloons in unselected, all-comers patients including also challenging lesion morphologies such as chronic total occlusions (CTO) and acute coronary syndromes.

## Methods

### Investigational device

**Fig. 1:**
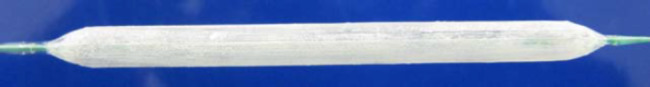
Crystalline Sirolimus-coated balloon catheter (cSCB, SeQuent® SCB, B.Braun Melsungen AG) with a dose of 4 µg/mm^2^ (crystalline sirolimus coating) [25]

The POBA platform is coated with crystalline sirolimus (Fig. 1) with a dose of 4 µg per mm^2^ balloon surface using a butylated hydroxyl toluene as an excipient. The exact composition is proprietary.

### Trial design

**Fig. 2:**
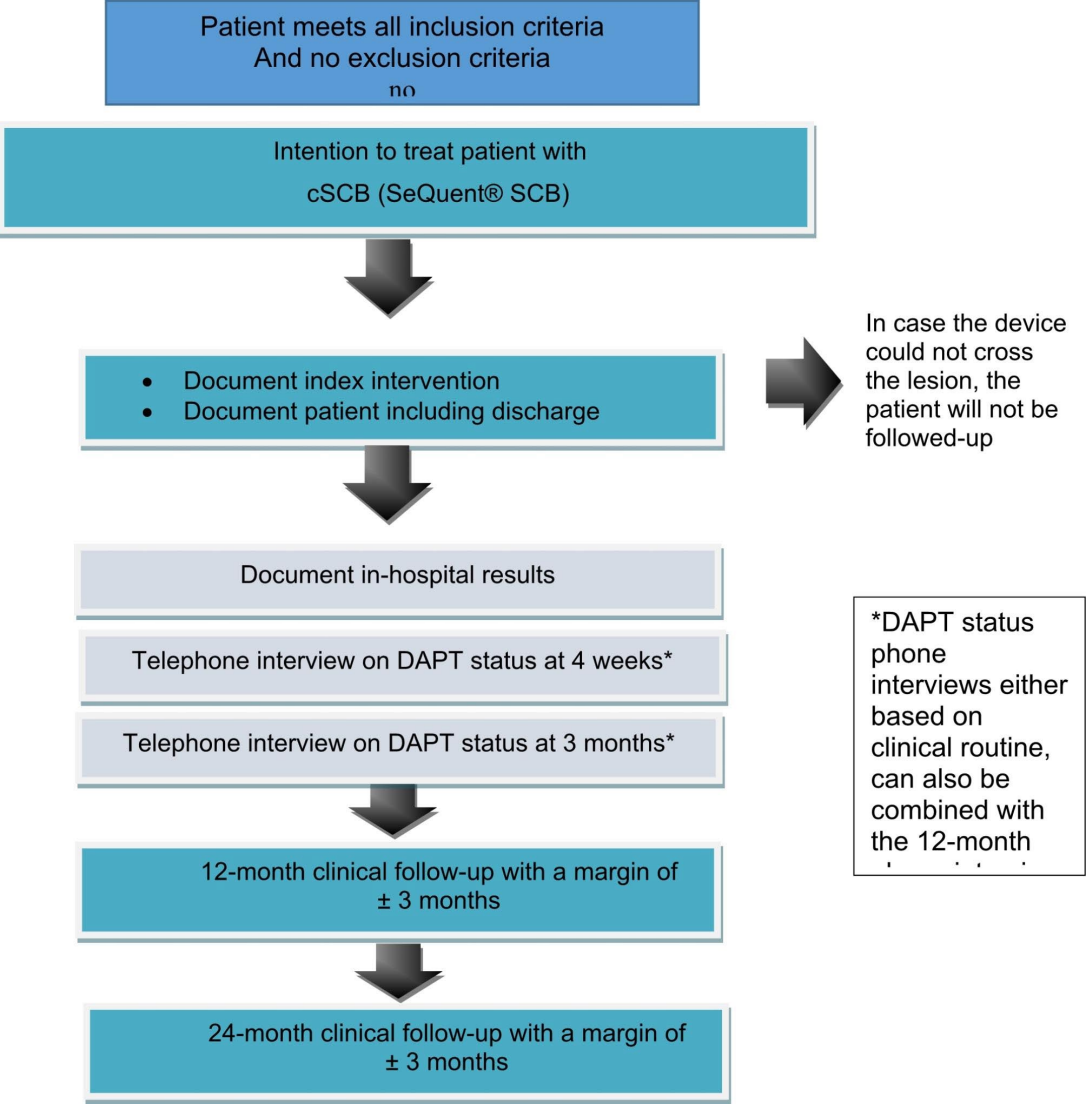
Flow-chart of Patient Recruitment and Planned Diagnostic Procedures

This is a single-armed, prospective, international, multi-center, post-market study in patients with coronary artery disease and indication for percutaneous coronary intervention (PCI) either due to documented ischemia by non-invasive or invasive functional testing or due to angina symptoms and a relevant stenotic coronary lesion during angiography (Fig. 2). The aim of the study is to assess continued safety and efficacy of the cSCB. The product under investigation will be used in routine clinical practice according to the latest ESC guidelines and according to the instructions for use. Those data that are obtained in routine clinical use will be documented in the Case Report Form (CRF). All patients who undergo a target intervention with the SCB will be followed for 12 and 24 months after initial PCI.

### Objectives

The objective of the study is to assess the safety and efficacy of crystalline sirolimus-coated balloon (cSCB, SeQuent® SCB, B.Braun Melsungen AG) within its approved indications to treat “real world” de-novo and restenotic lesions in native coronary arteries and coronary bypass grafts.

### Aim

The aim of the study is to assess the safety and efficacy of the cSCB to treat coronary artery disease with reference vessel diameters between ≥ 2 mm and ≤ 4 mm with suitable lesion lengths. There is no limitation of lesion lengths. In case the lesion is longer than 36 mm, more than one device needs to be used.

## Statistics

### Primary outcome variable

The primary outcome variable is the target lesion failure (TLF) rate at 12 months, defined as the composite rate of target vessel myocardial infarction (TV-MI), cardiac death or ischemia-driven target lesion revascularization (TLR).

### Secondary outcome variable

The secondary outcome variables are listed as follows:

- TLF at 24 months.
- Ischemia driven TLR at 12 and 24 months.
- All-cause death, cardiac death at 12 and 24 months.
- All myocardial infarction and TV-MI at 12 and 24 months.
- Major adverse coronary event (MACE), defined as composite of cardiovascular death, myocardial infarction or ischemia-driven TLR at 12 and 24 months.
- Duration of DAPT in real-life with (telephone) follow-up at 4 weeks, 3 and 12 months.
- Probable or definite stent-thrombosis of In-stent treated lesions accumulated at 12 and 24 months.
- Procedural success (final diameter stenosis < 30% without flow-limiting dissections).
- Major and minor bleeding complications according to BARC classification, during hospital stay and accumulated at 3, 12 and 24 months follow-up. Severe bleeding is defined as categorized with BARC 3–5.

### Sample size

Even though this is a single-armed, observational study within clinical routine (post-market surveillance), a pro-forma sample size calculation was highly recommended by the Notified Body of the manufacturer. In particular, the predecessor device iopromide-paclitaxel coated DCB is suited as a comparator.

The target lesion failure rate (TLF) is chosen as the primary variable. The study is designed to detect a difference in TLF in this patient population as compared to comparable patient groups described in the literature, i.e. the test hypotheses are:

#### H_0_

TLF_cSCB_ ≥ TLF_PCB_.

#### H_a_

H_0_ is false or TLF_cSCB_ < TLF_PCB_.

The target lesion failure rate in cSCB is not higher than or equal to the historic and published TLF rate of the predecessor device PCB.

Based on the above-mentioned hypothesis we assume the following:

Alpha = 5%.

TLF_PCB_= 6.8% (historic group from the literature, [15])

TLF_cSCB_= 6.8% (test group).

Follow-up rate = 85%.

Non-inferiority margin = 3.0%.

Based on the above assumptions 1106 patients must be followed up to compare their TLF rate to the TLF rate of the predecessor device. To account for patients lost to follow-up, a follow-up rate of 85% from previous studies will be assumed so that a total of 1302 patients will have to be recruited.

### Pre-specified analyses

Additionally, the following definitions are made:

- Patients who withdrew from this study are not replaced by additionally recruited patients to meet the minimum target of 1302 patients.
- nQuery Advisor® 7.0 software was used for sample size calculations.
- If a study site is recruiting less than 10 patients with less than 50% of follow-up, the patient data will be excluded from the analysis unless there are AE reported in this site.
- If the target number of patients are reached prior to the expected end of the recruitment period, patient enrolment will continue to the specified last day of recruitment.
- In case the necessary number of patients are not reached within the defined recruitment time, an extension for patient enrolment is possible.

All data will be analyzed by means of tables, figures, listings and statistical tests if appropriate. The final programming will be performed after closure of the database by use of an appropriate statistical software package (e.g., SPSS, SAS or R).

Since there is a wealth of clinical data for PCB angioplasty based on the all-comers approach, a propensity-score (PS) matched analysis is planned to compare SCB and PCB treatment of de novo and different types of ISR. PS matching will be done with a dependent variable adequately representing lesion complexity while focusing on de novo lesions only. Given a substantially sized clinical data base based on paclitaxel-coated balloon angioplasty with a similar electronic case report form, the relationship of clinical events and lesion morphologies can be investigated.

### Prespecified subgroups

Dedicated subgroup analyses include all patients who were treated for chronic total inclusions (CTO), the Asian study population and other groups with a higher than expected clinical outcome rate such as diabetics and patients of older age (≥ 75 years).

### Interventions

Patients with indication for PCI according to current guidelines doi: 10.1093/eurheartj/ehy394 are suitable for study participation. Estimation of %vessel stenosis, vessel diameter, stenosis length, lesion morphology, and if needed functional assessment using FFR or related indices (iFR, RFR) or intravascular imaging are done by operators’ discretion. Adequate lesion preparation with pre-dilation using semi- and/or non-compliant balloons with a balloon-to-vessel ration of 1:1 or debulking devices is performed on operator’s discretion. It is encouraged to achieve a residual diameter stenosis of ≤ 30% before dilating the lesion with the cSCB balloon (SeQuent SCB, B Braun Melsungen GmbH, Germany) according to the manufacturer IFU. Short delivery time of the SCB, slow balloon inflation, sufficient inflation time (30–60 s) and a balloon/vessel ratio of 1:1 or slightly higher should be attempted. In case of suboptimal angiographic results after lesion preparation (flow-limiting dissections, residual stenosis > 30%, FFR ≤ 0.80, TIMI-flow grade ≤ 2) bail-out stenting using a modern drug-eluting stent is advised [22]. The lesion and at least 2 mm proximally and distally should be covered with the DCB. For long lesions > 36 mm two DCBs have to be used. For bifurcation lesions, both, lesions of the main branch (MB) and the side branch (SB), are feasible for treatment with the study device. In bifurcation lesions 2 DCB strategies can be used by the operator: (1) DES in MB and DCB in SB, or (2) DCBs in both, MB and SB. The electronic CRF will cover the use of rotational atherectomy in the lesion treatment section along with other lesion preparation strategies.

Based on prior data [16], the most reliable indicator is the BASKET SMALL 2 trial which randomized after predilation similar to the SCORE screening protocol. Jeger et al. reported that 14.2% of all screened patients were amenable to stenting due to recoil or dissections which required permanent vessel support.

### Evaluation of adverse events

An independent critical event committee consisting of three members from Denmark (Prof. Jens Lassen, Odense Universitetshospital & University of Southern Denmark), Germany (Dr. Florian Krackhardt, Charité Universitätsmedizin, Berlin) and France (Dr. Georgios Sideris, Georges Pompidou APHP Paris) evaluate all device-event relationships for serious adverse events.

Adverse events (AE), including non-serious and serious AEs, are continuously monitored before patient discharge of index PCI and during 4-weeks, 3-, 12- and 24 months follow-up.

### Stopping and Discontinuation Criteria

The study will be stopped if the in-hospital TLR rate in the first 50 patients is higher than 10% and/or the in-hospital MACE rate is higher than 15%. At any rate, all included patients will be followed-up according to the protocol.

## Recruitment

### Patient inclusion criteria

- All common significant coronary lesions with clinical indication for PCI of a de novo or in-stent coronary stenosis according to the latest ESC guideline [11] recommendations.
- Any target lesion length > 36 mm needs to be covered with at least 2 devices.
- Patients eligible for this study must be at least 18 years of age.
- The patient must fulfil the standard recommendations for PCI, based on the last ESC recommendations within his/ her regular treatment or that the use of the product has already been decided within the regular planning of the patient’s treatment.
- In patients with multi-vessel coronary artery disease all vessels other than the target-vessel will be treated according to the operator’s discretion.
- In case more than one vessel is treated with the investigational device (cSCB) all vessels will be separately documented and analyzed.
- Only one lesion per vessel shall be included.
- In case more than one lesion need to be treated in the target-vessel, all lesions treated in a different way than the investigational procedure must be separated from the target-lesion by ≥ 20 mm or shall be seen as one lesion treated according to this study protocol.
- Written informed consent.

### General patient exclusion criteria

- Known intolerance to sirolimus.
- Allergy to any component of the coating.
- Severe allergy to contrast media.
- Pregnancy and lactation.
- Hemorrhagic diathesis or another disorder such as gastro-intestinal ulceration or cerebral circulatory disorders, which restrict the use of platelet aggregation inhibitor therapy and anti-coagulation therapy.
- Cardiogenic shock.
- Patients with an ejection fraction of < 30%.
- Comorbidity with a life expectancy < 1 year.
- Contraindication for whichever accompanying medication is necessary.
- Treatment shortly after myocardial infarction with indications of thrombus or TIMI flow ≤ 2.
- Indication for surgical revascularization.

### Angiographic exclusion criteria

- Complete occlusion of the target vessel. Complete occlusions present an exclusion criterion for the majority of the participating study sites. However, in preselected centers with expertise in CTO interventions and a proven track record (> 10% of all DCB interventions) of DCB application in CTO cases, complete occlusions may still be treated if there is a sufficiently high benefit-risk-ratio due to operators discretion.
- Lesions which are untreatable with PCI or other interventional techniques and coronary artery spasm in the absence of a significant stenosis.
- Vascular reference diameter < 2.00 mm or > 4 mm.
- Treatment of the left main coronary artery as study lesion.
- Target lesion not suitable for a drug-coated balloon-only PCI based on the discretion of the operator (e.g. severe calcification, subtotal occlusion).

QCA will be done by a central core lab with a published track record in interventional cardiology (University of Aarhus, Denmark).

## Data Availability

The datasets generated and/or analysed during the current study are not publicly available due intellectual property related restrictions but are available from the corresponding author on reasonable request. Since this is a post-market clinical follow-up study by nature, all study materials i.e., drug-coated balloons were not available free of charge as mandated by local laws.
